# Diffusion of Shape Stabilized PEG-SiO_2_ as a Driver for Producing Thermoregulating Facing Bricks

**DOI:** 10.3390/ma14061395

**Published:** 2021-03-13

**Authors:** Angel Serrano, Ana M. Borreguero, Isabel Iglesias, Anselmo Acosta, Juan F. Rodríguez, Manuel Carmona

**Affiliations:** 1 Centro de Investigación Cooperativa de Energías Alternativas (CIC energiGUNE), Basque Research and Technology Alliance (BRTA), Parque Tecnológico de Alava, Albert Einstein 48, 01510 Vitoria-Gasteiz, Spain; aserrano@cicenergigune.com; 2 Institute of Chemical and Environmental Technologies, Department of Chemical Engineering, University of Castilla—La Mancha, Av. Camilo José Cela s/n, 13004 Ciudad Real, Spain; anamaria.borreguero@uclm.es (A.M.B.); manuel.cfranco@uclm.es (M.C.); 3 Department of Mineralogy and Crystallography, University of Castilla—La Mancha, Av. Camilo José Cela s/n, 13004 Ciudad Real, Spain; Isabel.Iglesias@uclm.es (I.I.); Anselmo.Acosta@uclm.es (A.A.)

**Keywords:** facing bricks, shape-stabilized PCMs, polyethylene glycol, diffusion coefficient, sol-gel, thermal energy storage

## Abstract

A novel form-stable phase-change material (PCM) based on facing bricks was developed by incorporating thermoregulating PEG-SiO_2_, synthetized by sol-gel method and based on polyethylene glycol as phase-change material and silica as stabilizer compound. The PEG-SiO_2_ in its liquid form (*sol*) is firstly adsorbed inside the porous brick and lastly stabilized (*gel*) by controlling its gelation time, obtaining form-stable PCMs with PEG-SiO_2_ contents within 15–110 wt.%. Kinetic adsorption curves of the *sol* into bricks having different porosities as well as maximum adsorption capacities were obtained. The effective diffusion coefficients (*D_eff_*) were estimated by means of Fick’s second law, it being possible to predict the adsorption of *sol* PEG-SiO_2_ by the brick as function of its porosity and the free diffusion coefficient. Finally, form-stable PCMs demonstrated an improvement in their thermal energy storage capacity (up to 338%), these materials being capable of buffering the indoor temperature during an entire operational day

## 1. Introduction

Building energy efficiency is becoming a priority for global sustainable development since they represent 40% of the European Union’s final energy consumption [1]. Most of their energy expenditure can be reduced by applying thermal energy storage (TES) systems. Latent heat thermal energy storage system (LHTES) is one of the TES methods that has raised more interest in the last decades [2]. LHTES is carried out by using a convenient phase-change material (PCM). Three different types of PCMs are currently known: liquid–gas [3], liquid–solid [4], and solid–solid [5,6] PCMs, absorbing and releasing a large amount of heat during their phase-change process [7]. LHTES exhibits a higher energy density compared to any other thermal energy storage method at the time that reduces the energy losses derived from the temperature variation [8].

Different strategies have been studied for the application of PCMs for LHTES in buildings, since they are easily adsorbed into porous concrete, gypsum, or in the polyurethane foams matrix, thus achieving successful LHTES systems suitable for building facades [9,10,11,12]. However, despite bricks being one of the oldest and most used building materials in history [13], there is a lack of studies for improving their thermal storage capacity. To date, one of the main current approaches focuses on the use of PCMs in filling the voids of hollow bricks [14,15]. This approach demonstrated that the PCMs addition reduced the temperature fluctuations and the speed of the temperature change in the indoor space [16,17]. Nevertheless, the loss of lightness (main characteristic of hollow bricks) and the use of complementary elements to avoid the leakage of the PCM, which increases the cost of the material, could limit its implementation.

The second most common method to combine PCMs with bricks is based on the use of additional layers added onto the outer brick face of the walls or as a middle layer, mainly by means of shape-stabilized phase-change materials (ssPCMs) [18,19,20,21,22]. Zhou et al. [23] numerically studied the combination of an inner layer of ssPCM with night ventilation. This additional coating was formed by the dispersion of PCM in high-density polyethylene (HDPE). Their results indicated that the use of both systems, PCM and night ventilation, could save 76% of cooling energy consumption. Despite the achieved improvements, the use of additional layers increases the wall thickness, and it could bring negative consequences like overly high airtightness, over-heating, poor ventilation, and loss of space [24].

With these premises, the use of facing bricks containing PCMs applied in the façade of buildings is an alternative option to enhance their energy efficiency. Due to the brick-firing process, PCM must be added once the bricks are completely manufactured. The small pore size of the facing bricks requires the addition of PCM in liquid state, by immersion or vacuum impregnation techniques. These methods are accomplished by the submersion of porous building elements (concrete, gypsum panels, wood, porous aggregates, etc.) into molten PCM, which is adsorbed by capillary action [25].

The materials produced by these techniques are known as form-stable PCMs. In contrast to ssPCMs, the production of form-stable PCMs does not require the melting or blending of the supporting material [26,27,28].

Most of the reported studies related to form-stable PCMs are mainly carried out using powder as supporting matrix instead of building blocks. Silica fume (particle size < 0.1 μm) [29], diatomite or expanded perlite (particle size < 150 μm) [30,31,32], expanded graphite (particle size between 50 and 500 μm), and gypsum or cement (particle size < 150 mm) [33] are some of the supporting matrices studied to date. 

The use of porous facing bricks could be affected by the leakage of the common solid–liquid PCM from the brick when it turns into liquid. In order to solve these problems, a previously developed PEG-SiO_2_ ssPCM by sol-gel is proposed to be incorporated in bricks [34]. A similar strategy has been previously employed by Xu et al. (2020) [35] focused on porous wood matrices. In their work, they used a commercial silica sol with a solid content of 30%, an average particle size of 15 nm, and a pH value of 10 to stabilize the polyethylene glycol into the porous wood. The incorporation of the PEG was carried out by using a vacuum-pressure process at −0.1 MPa. Once impregnated, the samples were cured in an oven at 60 °C for 2 days.

Nevertheless, to date there are no studies that apply this approach to facing bricks production containing PCMs, despite the great advantages of combining the thermal properties of these two materials. Likewise, the adsorption of *sol* materials into porous bricks for further synthesis of ssPCMs by sol-gel method has not previously been addressed. Thus, there is a lack of knowledge about the effect of the viscosity increase with time in the adsorption process (kinetic and distribution) and about its possible theoretical modelling.

In this work, the stabilizer material of the ssPCM is based on our own development of PEG-SiO_2_. In this case, the silica network is in situ synthesized from an alkoxide precursor, tetraethyl orthosilicate (TEOS), during its mixture with the PCM (PEG) in the *sol* formation step. This *sol* can be turned into *gel*, controlling the pH value of the medium as described in the previous work [34]. Hence, the PEG-SiO_2_ mixture can be incorporated into the porous ceramic material in its *sol* state by capillary action, before being stabilized by controlling its gelation time.

The PEG-SiO_2_ rheology is analyzed by studying the evolution of the *sol* viscosity with time and determining the point in which the *sol* rheological behavior starts to change significantly. This point is interesting for designing the immersion experiments. Bricks with porosities between 0.44 and 0.77 were used to study the influence of porosity in the adsorption kinetic and in the maximum adsorption capacity at 50 °C. The unknown effective diffusion coefficient of PEG-SiO_2_ into the porous bricks was estimated by means of Fick´s second law, using the orthogonal collocation as strategy for solving the partial differential equation. The use of a unique free diffusion coefficient for modelling the diffusion process for the different studied porosities was also considered.

## 2. Experimental

### 2.1. Materials

Polyethylene glycol of molecular weight 950–1050 g/mol (PEG1000), tetraethyl orthosilicate (TEOS) (98%), and sulfuric acid (95–97%) were purchased from Sigma Aldrich (Madrid, Spain). Ethanol (EtOH) (96%) and sodium hydroxide (NaOH) (98%) were supplied by PANREAC. Common clays from Santa Cruz de Mudela, Spain, traditionally used for the industrial production of bricks and roof tiles, have been provided by Rústicos La Mancha S.L., Santa Cruz de Mudela, Spain, and used for the brick production.

### 2.2. Bricks and Porosity

Five bricks were manufactured, controlling their porosity by adding specific percentages of biomass from the wood industry together with clay, resulting in a homogenous paste. This paste was molded and baked at 940 °C, obtaining bricks of 10 × 6 × 3 cm^3^ with a porosity related to the quantity of added biomass [36]. Table 1 shows the final porosity (ε) of the bricks, obtained by helium pycnometry.

### 2.3. PEG-SiO_2_ Synthesis

PEG-SiO_2_ was synthesized by sol-gel method. PEG1000 exhibits a melting point of 35 °C, crystallization temperature of 27 °C, and a latent heat of 145 J/g. In a first step, the polyol was melted and heated up to 50 °C, the rest of the compounds were subsequently added to the liquid polyol setting a molar ratio H_2_O:EtOH:H_2_SO_4_:PEG1000:TEOS of 2:0.34:0.021:0.50:1 and stirred for 30 min. The temperature was controlled at 50 °C in a constant temperature bath. The mixture obtained using the commented procedure is denominated as *sol*. To obtain the final PEG-SiO_2_, the *sol* will need to be gelled by changing the pH of the medium once it is inside the porous matrix of the brick. Thermal properties of the synthesized PEG-SiO_2_ when it is gelled in absence of the brick are a melting temperature of 35.22 °C and latent heat of fusion (∆*H*_m_) of 113.80 J/g, crystallization temperature of 22.94 °C, and crystallization heat (∆*H*_c_) of 105.60 J/g.

### 2.4. Form-Stable PCM Synthesis

**Figure 1:**
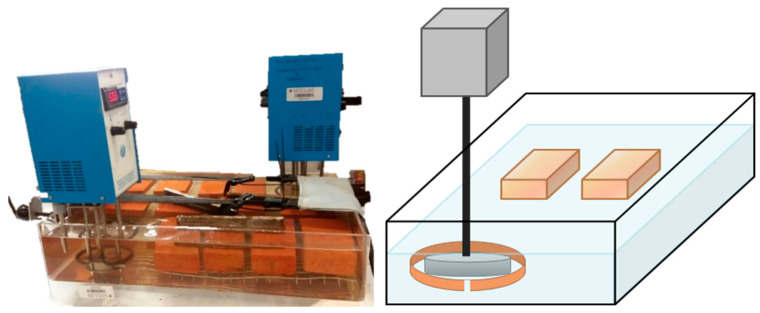
Scheme of the immersion system for the preparation of the form-stable phase-change material (PCMs).

Figure 1 shows the immersion device for accomplishing the incorporation of PEG-SiO_2_ in bricks. The setup consists of a methacrylate container with dimensions 60 × 50 × 10 cm^3^ equipped with a thermostatic bath (Mervilab, Madrid, Spain) and a stirrer (Mervilab, Madrid, Spain)). A metal mesh was installed at the bottom of the container to avoid contact of the bricks with the methacrylate.

During the immersion process, the temperature was set at 50 °C. The high specific heat of the water/PEG mixture (*sol*) ensured a constant temperature during the whole process. In addition, the low water concentration in the mixture (≈7 wt.% with respect to PEG) combined with the hygroscopic properties of PEG prevented water loss during the test.

The bricks were immersed in the *sol* mixture and taken out at the desired times. After this process, by means of a brush, the samples were smeared with a NaOH solution (5 M) to promote the gelation of the *sol* inside the porous matrix of the bricks. With this brushing, we obtained an excess of NaOH that promoted a fast gelation (the increase of viscosity was evident at simple sight). Finally, once this fast gelation had occurred, the aging of the *gel* was carried out in an oven for 24 h at 50 °C, the moment in which the *gel* dried by shrinkage and the PEG-SiO_2_ was adsorbed by the brick structure.

### 2.5. Characterization

#### 2.5.1. Sol Viscosity (Rheology)

The evolution of the *sol* viscosity with time was followed through a rheological study. The viscosity was obtained in a Bohlin Gemini rheometer (Malvern, Worcestershire, UK) at 50 °C, using a cone (CP 1°/60) with 60 mm of diameter and a share rate from 15 to 1570 s^−1^, recording the data every 10 s.

#### 2.5.2. Adsorption Curves and Maximum Adsorption Capacity

The kinetic adsorption curves and the maximum adsorption capacity (*C*_e_) of the bricks by immersion technique were studied. During the form-stable PCM production the different porous bricks were removed from the *sol* mixture at different times. Each sample was weighted before and after the PEG-SiO_2_ incorporation, once the drying stage was finished. In addition, three bricks were spent for each adsorption time. For the maximum adsorption capacity the samples were immersed in the *sol* mixture until their mass was constant (around 72 h). This time was long enough to reach the filling of the porous brick with the *sol* without any significant change in the external *sol* viscosity.

#### 2.5.3. Bulk Density, True Density, and Porosity

The bulk density of the composites, ρ_bulk_, was quantified by weighing and sizing the test prisms. The matrix density or true density, ρ_comp_, was determined by helium pycnometry (Micromeritics Accupyc 1330). The porosity of the brick and gypsum composites, ε, (Equation (1)) was estimated from the bulk density, the matrix density, and assuming that their pores are filled with air. The air density (ρ_air_) was assumed to be 1.186 kg/m^3^ at normal conditions.

(1) $$\varepsilon =\frac{\rho_{\mathrm{comp}}-\rho_{\mathrm{bulk}}}{\rho_{\mathrm{comp}}-\rho_{\mathrm{air}}}$$

#### 2.5.4. Scanning Electron Microscopy Analysis

The inner structure of the obtained composites was analyzed by means of scanning electron microscopy (SEM, FEI Company, Madrid, Spain) by using a FEI QUANTA 200. Large-field low-vacuum detector (LFD, FEI Company, Madrid, Spain) was used to avoid the melting of the PCM during the study. In order to have a better representation of the PCM distribution within the structure itself, the samples were sectioned, studying the central part of their cross-section.

#### 2.5.5. Experimental Equipment for Thermal Behavior Tests

The thermal behavior of the composites has been studied using a homemade equipment composed of a hollow metallic box of aluminum through which a liquid coming from a thermostatic bath (Mervilab, Madrid, Spain) was flowing by means of a peristaltic pump. The temperature of this liquid allowed us to control the temperature on the aluminum cell. The sample blocks were placed on the upper surface of the cell and further insulated with foam boards of 3.9 cm in thickness. A more exhaustive description of the set-up and of its performance for the thermal characterization of materials containing PCMs was provided in previous works [37]. Tests were carried out applying a thermostatic bath set-point step change from 15 to 42 °C while temperatures at different sample locations and the inlet and outlet heat fluxes were registered with time. The dimensions of the samples were 10 × 6 × 3 cm^3^. Six thermocouples (TC Direct, Madrid, Spain) of K-type were located across the probe thickness: two in the external sample surface (*T*_up_), which would correspond to the building indoor temperature; two in the middle (*T*_middle_); and the other two on the aluminum cell (*T*_down_), which would correspond to the external temperature. The heat fluxes were measured by using gSKIN^®^-XI and gSKIN^®^-XP heat flow sensors (gSKIN, Rümlang, Switzerland) for monitoring the inlet and outlet heat fluxes. Using these signals and Equation (2), it is possible to quantify the TES capacity per cubic meter.

(2) $$TES=\frac{q_{acc}}{m_{sample}}\times \frac{\rho_{\mathrm{bulk}}}{3.6\times 10^{6}}$$

## 3. Calculations

### 3.1. Problem Definition for Diffusion Coefficients Calculation

To estimate the effective diffusion coefficients of the obtained composites, the diffusion process of the PEG-SiO_2_ (or *sol*) inside the porous bricks was thoroughly studied; for that purpose, the amount of total accumulated PEG-SiO_2_ was measured over time. The brick under discussion has a cubic geometry with *x*_o_ (10 cm), *y*_o_ (3 cm), and *z*_o_ (6 cm) as dimensions.

**Figure 2:**
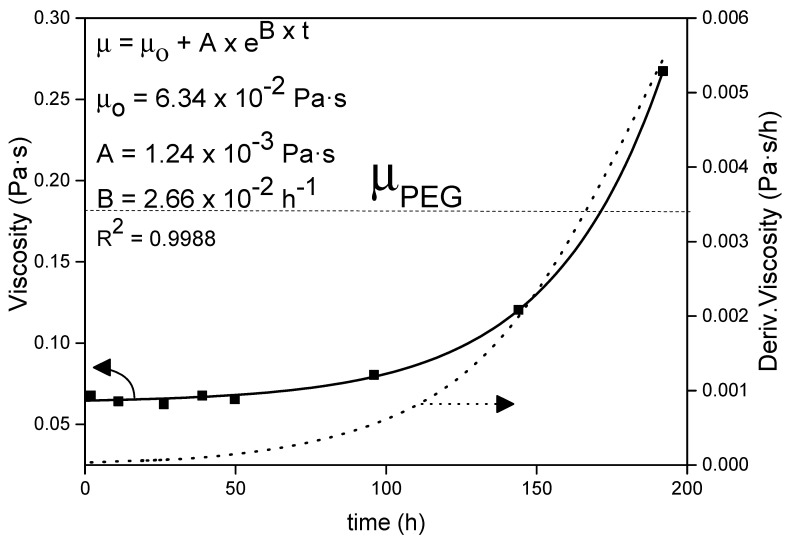
Evolution of the *sol* viscosity with time.

For the problem resolution of this three-dimensional coordinate system, some assumptions have been taken into consideration: (a) homogeneous distribution of the pores and their size through the whole brick; (b) the *sol* viscosity remains constant until the complete *sol* adsorption. This last assumption is corroborated by Figure 2, where the viscosity is observed with no significant changes for times shorter than 72 h.

Then, when the whole slab is immersed into the invariable *sol*, applying the mass balance across the volume control which is built around a set of three axes, with neither convection flux nor chemical reaction, Fick’s second law (Equation (3)) can be derived. This equation is frequently found in literature applied for the diffusion through solids when the diffusivity is independent of the concentration [38].

(3) $$\frac{\varepsilon \partial C_{A}}{\partial t}=D_{\mathrm{eff}}\times \left(\frac{\partial^{2}C_{A}}{\partial x^{2}}+\frac{\partial^{2}C_{A}}{\partial y^{2}}+\frac{\partial^{2}C_{A}}{\partial z^{2}}\right)$$

where ε is the solid porosity, *D_eff_* is the effective diffusion coefficient of A through the solid, and *C*_A_ is the concentration of the compound A inside the solid.

Dimensionless variables (Equation (4)) based on the maximum adsorption capacity (*C*_e_) and dimensions of the bricks can be defined in order to normalize the problem in the range from 0 to 1, turning Equation (3) into Equation (5).

(4) $$\frac{C_{A}}{C_{e}}=C;\;\frac{x}{x_{o}}=X;\;\frac{y}{y_{o}}=Y;\frac{z}{Z_{o}}=Z;\;\tau =\frac{t\;\times \;D_{\mathrm{eff}}}{\varepsilon \;\times \;x_{0}^{2}}$$

(5) $$\frac{\partial C}{\partial \tau }=\left(\frac{\partial^{2}C}{\partial X^{2}}+{\left(\frac{x_{0}}{y_{0}}\right)}^{2}\times \frac{\partial^{2}C}{\partial Y^{2}}+{\left(\frac{x_{0}}{z_{0}}\right)}^{2}\times \frac{\partial^{2}C}{\partial Z^{2}}\right)$$

### 3.2. Orthogonal Collocation Method in Fick´s Second Law

Orthogonal collocation method can be used to solve the above partial differential equation since this method is applied for problems whose solutions are regular and with absence of sharp changes. In the orthogonal collocation method, the exact solution is estimated through a trial function $\tilde{Y}.$ In that way, the solution is represented as a polynomial, *P(x)*, with unknown coefficients, *b*, which are determined at certain points where the trial function satisfies the process equation (Fick´s second law in this case), as well as the boundary and initial conditions (Equation (6)) [39]. The selected points, denominated as collocation points, minimize the estimation error.

(6) $$trial\;function=\tilde{Y}=\overset{N}{\underset{i=1}{\sum }}b_{i}\times P_{i}\left(x\right)$$

where *N* is the number of interior collocation points, which are the roots to *P*_N_(*x*) = 0. Some of the most typical roots for different number of collocation points and polynomials were listed by Finlayson (1972) [40].

For solving Equation (5), the second derivative of the trial function depending on the coordinate system and considering the geometry is required. This second derivative can be written as shown in Equation (7).

(7) $$x^{1-a}\frac{d}{dx}\left(x^{a-1}\frac{d\tilde{Y}}{dx}\right)=\frac{d^{2}\tilde{Y}}{dx^{2}};being\;a=1\;for\;planar\;geometry$$

Considering Equations (6) and (7), deriving and evaluating in each collocation point, the above equation can be expressed as:

(8) $${\frac{d^{2}\tilde{Y}}{dx^{2}}|}_{x_{j}}=\overset{N+1}{\underset{i=0}{\sum }}B_{m\;j,i}\times {\tilde{Y}}_{i};\;j=0,\ldots ,N$$

where *B_m_* matrix represents the Laplacian at the collocation points.

The first boundary condition is defined at the center of the object,

(9) $${\frac{d^{2}\tilde{Y}}{dx^{2}}|}_{x_{j}}=\overset{N+1}{\underset{i=0}{\sum }}B_{m\;j,i}\times {\tilde{Y}}_{i};\;j=0,\ldots ,N$$

where the *A_m_* matrix represents the first derivative at the collocation points.

The second boundary condition is defined at the external surface of the object,

(10) $${\frac{d\tilde{Y}}{dx}|}_{x=1}=-\overset{N+1}{\underset{i=0}{\sum }}A_{m\;N+1,i}\times {\tilde{Y}}_{i}=Bi_{m}\left(Y^{*}-{\tilde{Y}}_{N+1}\right)$$

where Y* is the solid concentration at the equilibrium with the concentration of the bulk solution and *Bi_m_* is a Biot number for mass transfer which is defined by:

(11) $$Bi_{m}=\frac{k\times x_{0}}{D_{eff}}$$

where *k* is the constant of mass transfer and *x*_0_ is the size of the object in the *x* dimension.

For an unsteady-state problem, as the current case, the expression 5 is solved at any time t in three dimensions, ${\tilde{Y}}_{i}\left(t\right)=\tilde{Y}\left(x_{i},y_{i},z_{i},\;t\right)$ and at any collocation points *x_i_*, *y_i_*, *z_i_* [41].

Therefore, the diffusion of the *sol* through the brick expressed by the partial differential equation, Equation (5), having the Laplacian form for each dimension *X, Y, Z* can be transformed into a set of (NxNxN) total differential ordinary equations from τ.

(12) $$\frac{\partial C_{i,k,l}}{\partial \tau }=\left(\overset{N+1}{\underset{j=0}{\sum }}B_{m\;i,j}C_{j,k,l}+{\left(\frac{x_{0}}{y_{0}}\right)}^{2}\times \overset{N+1}{\underset{j=0}{\sum }}B_{m\;k,j}C_{i,j,l}+{\left(\frac{x_{0}}{z_{0}}\right)}^{2}\times \overset{N+1}{\underset{j=0}{\sum }}B_{m\;l,j}C_{i,k,j}\right)\{\begin{array}{c} i=1,\ldots N\\ k=1,\ldots N\\ l=1,\ldots N \end{array}$$

Using asymmetric geometry, and defining orthogonal points within 0.0–1.0, it is possible to avoid the use of the first boundary condition at the center of the block (*X = Y = Z* = 0.5) (*dC/dX = dC/dY = dC/dZ = 0*) which is required when the symmetry geometry is considered. Attending to the *sol* characteristics, its composition in the liquid phase does not change over time, the tank is perfectly agitated during the mass transfer limiting step located inside the brick, it being possible to assume that the external slab face is instantaneously saturated with sol (Y* ≅ *Y*_0_ = *Y*_*N* + 1_). Hence, the above set of differential equations can be solved considering that at the boundary points *X, Y*, or *Z* having values of 0 or 1.0 (being the counter *j* equal to 0 or *N* + 1 for each summation term) the dependent variables *C* are equal to 1.0. This is,

(13) $$C_{j,k,l}=1.0\;\left\{\begin{array}{l} j=0\;\mathrm{or}\;N+1\\ k=0,\ldots ,\;N+1\;\\ l=0,\ldots ,\;N+1 \end{array}\right\}\;;\;X\;=\;0.0\;\mathrm{or}\;X\;=\;1.0$$

(14) $$C_{i,j,l}=1.0\;\left\{\begin{array}{l} j=0\;\mathrm{or}\;N+1\\ i=0,\ldots ,\;N+1\;\\ l=0,\ldots ,\;N+1 \end{array}\right\}\;;\;Y\;=\;0.0\;\mathrm{or}\;Y\;=\;1.0$$

(15) $$C_{i,k,j}=1.0\;\left\{\begin{array}{l} j=0\;\mathrm{or}\;N+1\\ i=0,\ldots ,\;N+1\;\\ k=0,\ldots ,\;N+1 \end{array}\right\}\;;\;Z\;=\;0.0\;\mathrm{or}\;Z\;=\;1.0$$

Considering the above boundary conditions, it is possible to obtain the variation of the *sol* concentration into the internal nodes by using Rosenbrock method as standard technique for leading the numerical integration of the above set of (*N* × *N* × *N*) total differential ordinary equations. The integration begins with considering the brick initially free of *sol* which implies that *C* is equal to zero for each internal node and *D_eff_*/ε being the unique unknown parameter.

Hence, the derivative expression 10 becomes a constraint that must be satisfied by the solution of Equation (12) at each τ units, in order to reach the proper quantification.

(16) $${\frac{dC}{dX}|}_{X=0.0}={\frac{dC}{dY}|}_{Y=0.0}={\frac{dC}{dZ}|}_{Z=0.0}=0=-\overset{N+1}{\underset{i=0}{\sum }}A_{0,i}C_{i}$$

(17) $${\frac{dC}{dX}|}_{X=1.0}={\frac{dC}{dY}|}_{Y=1.0}={\frac{dC}{dZ}|}_{Z=1.0}=0=-\overset{N+1}{\underset{i=0}{\sum }}A_{N+1,i}C_{i}$$

## 4. Results and Discussion

### 4.1. PEG-SiO_2_ sol Rheology

The adsorption of a liquid by a porous matrix is promoted by the fluidity of the compound through the supporting material. In that way, the viscosity of the liquid compound plays a crucial role in its incorporation into the matrix by capillary forces. Figure 2 shows the evolution of the *sol* viscosity with time.

As can be observed, the viscosity of the *sol* shows the typical exponential growth with time presented by sol-gel products. This growth is due to the internal condensation that takes place between the polymers formed during the first step of the sol-gel process, finishing when they form a rigid gel structure, a moment known as gelation time [42]. The derivative with respect to the time of the viscosity growth expression (Equation (18)) provides information on the rate of change of viscosity with time.

(18) $$\frac{d\mu }{dt}=A\times B\times e^{B\times t}$$

In that way, according to Figure 2, the viscosity change is not very significant until about 72 h after its synthesis, having the *sol* display the same rheological behavior during that time. The final gelation time is higher than 200 h. Moreover, by changing the pH of the medium the gelation time can be controlled, since it has an inverse dependence with the reaction rate of the condensation step [43]. The control of the *sol* gelation time and the long period with constant rheological properties allow its incorporation into different porous materials. The *sol* can be adsorbed by the porous slabs (concrete, gypsum, bricks, wood, etc.) and further gelled in their inner structure. In addition, the infiltration of the young *sol* into the porous material is favored by its lower initial viscosity with respect to pure PEG1000, 0.06 Pa·s instead of 0.18 Pa·s, respectively, both measured at 50 °C.

### 4.2. Adsorption Capacity

**Figure 3:**
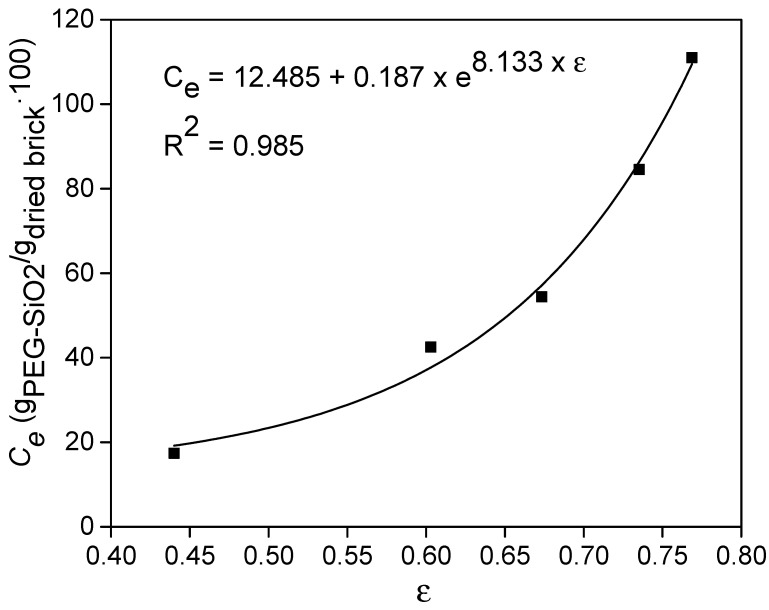
Maximum adsorption capacity for each porosity.

The maximum adsorption capacity (*C*_e_) of the bricks by immersion technique was studied as a function of their porosity. It was found that an equilibrium time of 72 h was long enough to reach the full filling of the porous brick with the *sol* without any significant change in the external *sol* viscosity. Figure 3 displays the maximum adsorption capacity as function of the porosity, since the external concentration does not change during the adsorption process. As expected, the higher the bricks’ porosity, the higher the adsorption capacity of the material. The equilibrium data can be fitted using different functions that relate *C*_e_ with the ε [44], being the curve perfectly fitted by an exponential function. The obtained equation can be used to predict the maximum amount of PEG-SiO_2_ that the brick is able to adsorb according to its porosity or for optimization tasks to calculate the required porosity of a tailored brick. It should be noted that the brick with the highest porosity can adsorb more than 110% of its dried mass, whereas the densest brick is only able to adsorb 20% of its weight. According to the figure, by changing the porosity it is possible to sharply increase the uptake of PEG-SiO_2_ by this clay.

### 4.3. Bulk Density

**Figure 4:**
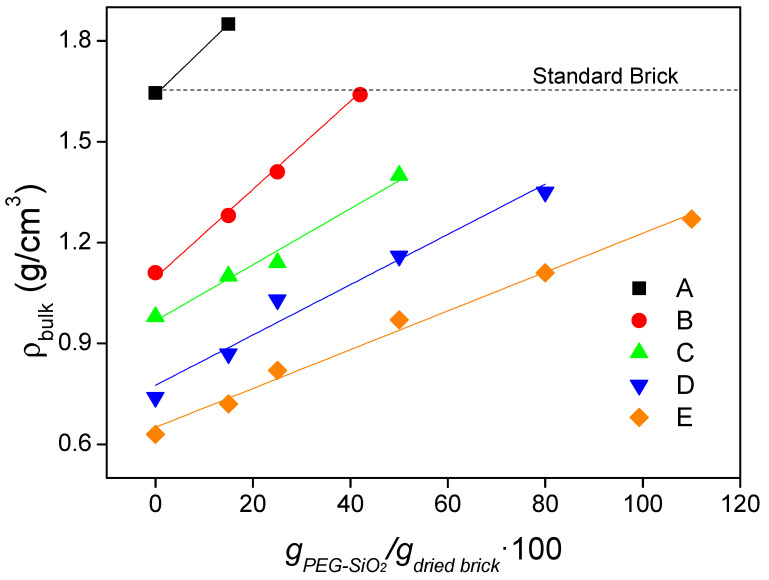
Bulk density of the composites as a function of the PEG-SiO_2_ content.

Figure 4 presents the evolution of the bulk density (ρ_bulk_) with the amount of incorporated PEG-SiO_2_ for the obtained composites. To obtain the different PEG-SiO_2_ additions, composites were taken out from the immersion bath at different times. As is evident from the results, the higher the PEG-SiO_2_ content, the higher the density of the composite, following a linear trend and without crossing between them, being practically parallel. These increments in the density are a consequence of the replacement of air by PEG-SiO_2_ after the immersion process. The composites containing the maximum amount of PCM do not exceed the density of the standard facing brick with no special treatment for increasing its porosity (brick type A).

### 4.4. SEM Analysis

**Figure 5:**
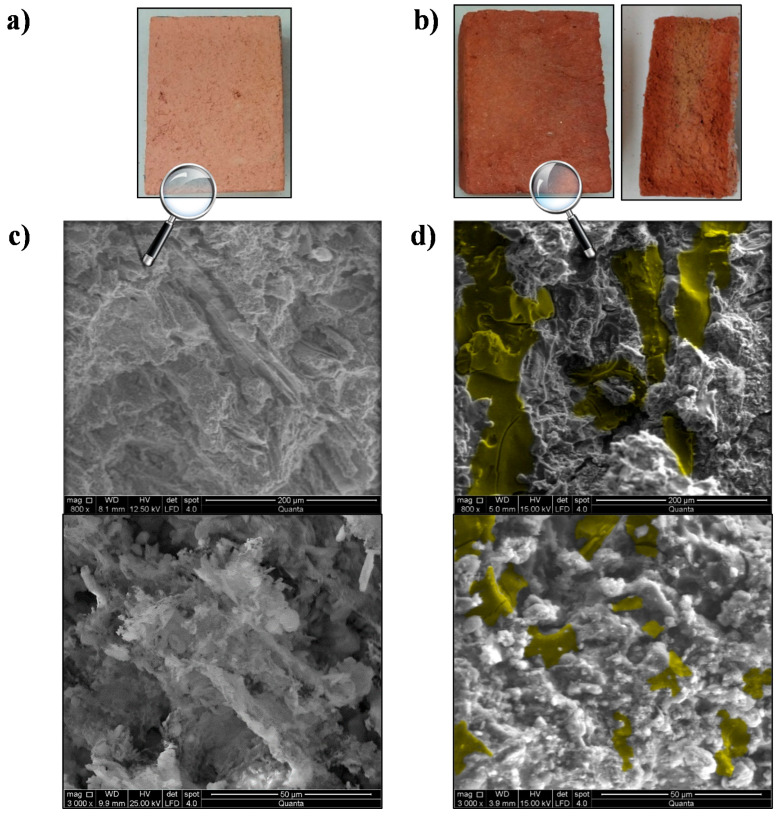
Visual appearance and scanning electron microscopy (SEM) images of D brick inner structure before (**a**,**c**) and after (**b**,**d**) the immersion process at different magnifications.

Figure 5 exposes the visual appearance and the SEM images at different magnifications of the inner structure of *D* brick before and after the described immersion process. The brick after immersion was cut to show its inner visual appearance (Figure 5b).

As can be seen in Figure 5b, the bricks after the immersion process presented darker color due to the presence of the PEG-SiO_2_. In addition, this darker color can be seen along the whole brick section.

The typical shape of the inner structure of a brick can be seen in Figure 5c, whose elemental composition was analyzed by EDAX. O (32.2 wt.%), Si (20.7 wt.%), Ca (14.3 wt.%), Fe (13.6 wt.%), Al (12.4 wt.%), Mg (5.2 wt.%), and K (1.6 wt.%) were the main elements present in the structure, ordered according to their amount in the brick. This composition is attributed to minerals such as dolomite, calcite, quartz, and feldspars, as well as clay minerals like illite or kaolinite, which are normally present in the bricks formed with the current type of clays [45]. In Figure 5d, PEG-SiO_2_ can be seen highlighted in yellow. As is evident from the picture, PEG-SiO_2_ is perfectly integrated in the internal structure of the brick, filling its pores and fissures and adopting the shape of its inner framework. In this case, the EDAX showed that the most common compound on the inner surface of the composite was carbon (C) (39.4 wt.%), followed by O (37.0 wt.%), Si (12.0 wt.%), Al (3.9 wt.%), Ca (3.7 wt.%), Fe (2.1 wt.%), Mg (1.2 wt.%), and K (0.7 wt.%). The emergence of C and its high presence together with O atoms confirm the distribution of the PEG-SiO_2_ in the whole composite.

### 4.5. Effective Diffusion Coefficients

**Figure 6:**
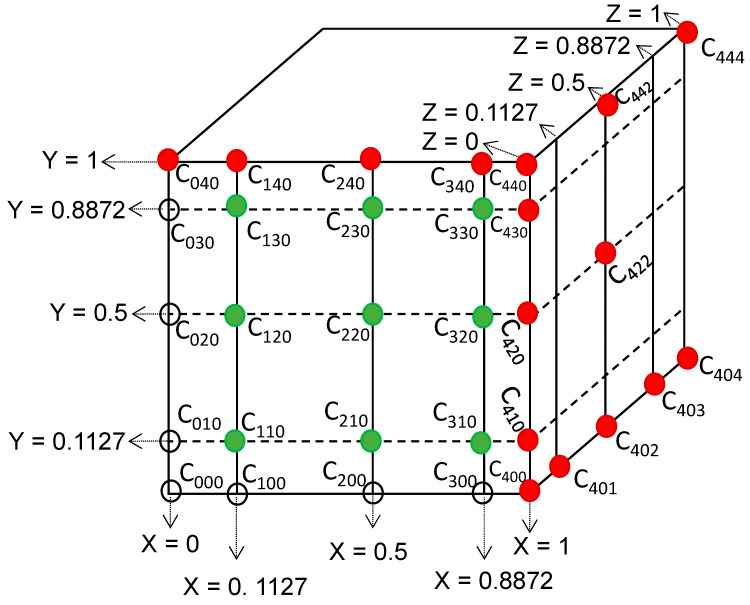
Distribution of some of the *N* internal collocation points and *N + 1* boundary nodes.

After verifying the presence of PEG-SiO_2_ inside the bricks, the estimation of the effective diffusion coefficient for each *sol*-brick system can be carried out according to Equation (12) and the conditions previously described (Calculations). For the resolution of the above partial differential equation, 3 internal collocation points were chosen (*N* = 3) for reducing the computation time. The distribution of some of the collocation points in the selected quadrant of the brick is represented in Figure 6 colored in green, whereas the boundary layer, *N* + 1, is represented in red color. Table 2 shows the value of *B_m_* matrix related with the 3 collocation points for a planar geometry and the considered polynomial, which can be found using the algorithm described by Villadsen and Stewart [41,46].

With the described equations, initial and boundary conditions, and assuming a value for the unknown parameter *D_eff_*/ε, the concentration (*C_i,k,l_*) of the *sol* in the brick on a scale from 0 to 1 can be calculated for each collocation point at different times, where 1 corresponds to the maximum adsorption capacity of the composite under consideration. As previously commented, the Rosenbrock method was used to numerically integrate the set of ordinary differential equations due to the few steps of integration required to achieve the solution [47]. Once all the *C_i,k,l_* values are obtained, the average theoretical value of *sol* concentration charged by the brick ($\bar{C}$) is calculated by means of Equation (12) at each time (*s*1).

(19) $$\bar{C_{s1}}=\frac{{\int \int \int }_{0}^{1}C_{i,k,l}dXdYdZ}{{\int \int \int }_{0}^{1}dXdYdZ}$$

In order to theoretically predict the value of the average concentration at each time ($\bar{C_{s1}}$) it is necessary to find the proper unknown parameter *D_eff_*/ε for each kind of brick that has perfect fit between the experimental ($\bar{C_{\exp\left(s1\right)}}$) and the theoretical values ($\bar{C_{s1}}$). To this purpose, Marquardt’s algorithm was used [48]. This method can be summarized by Equation (20).

(20) $$\Phi ={\left(\bar{C_{\exp\left(s\right)}}-\bar{C_{\left(s\right)}}\right)}^{T}\left(\bar{C_{\exp\left(s\right)}}-\bar{C_{\left(s\right)}}\right)$$

where Φ is the weighted sum of squared residuals, *s* is a counter from 1 to the number of experimental data, indicating the number of the sample taken out during the kinetic experiment at each time, and *T* is the transpose of the residual array.

A Visual Basic application was developed for solving this model.

**Figure 7:**
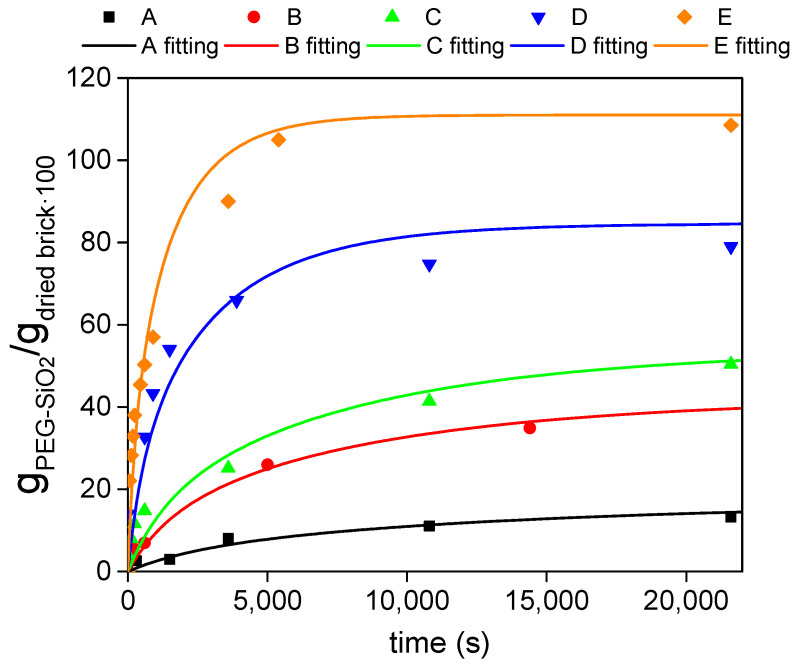
Experimental and modelled kinetic curves of PEG-SiO_2_ uptake by bricks having different porosities.

Figure 7 shows the kinetic experimental and modelled curves of the adsorbed PEG-SiO_2_ into dried bricks as a function of the porosity.

According to Figure 7, a good agreement between the experimental *sol* adsorption by the bricks and the modelled values was obtained. In addition, it is clear from the slope of the adsorption profiles that the higher the brick porosity, the faster the uptake of the *sol* by the brick. The obtained fitting parameter (*D_eff_*/ε) and their corresponding confidence intervals, using a confidential level of 95% (α = 0.05), are gathered in Table 3. As expected, bricks with the faster *sol* adsorption present the higher *D_eff_*/ε values.

**Figure 8:**
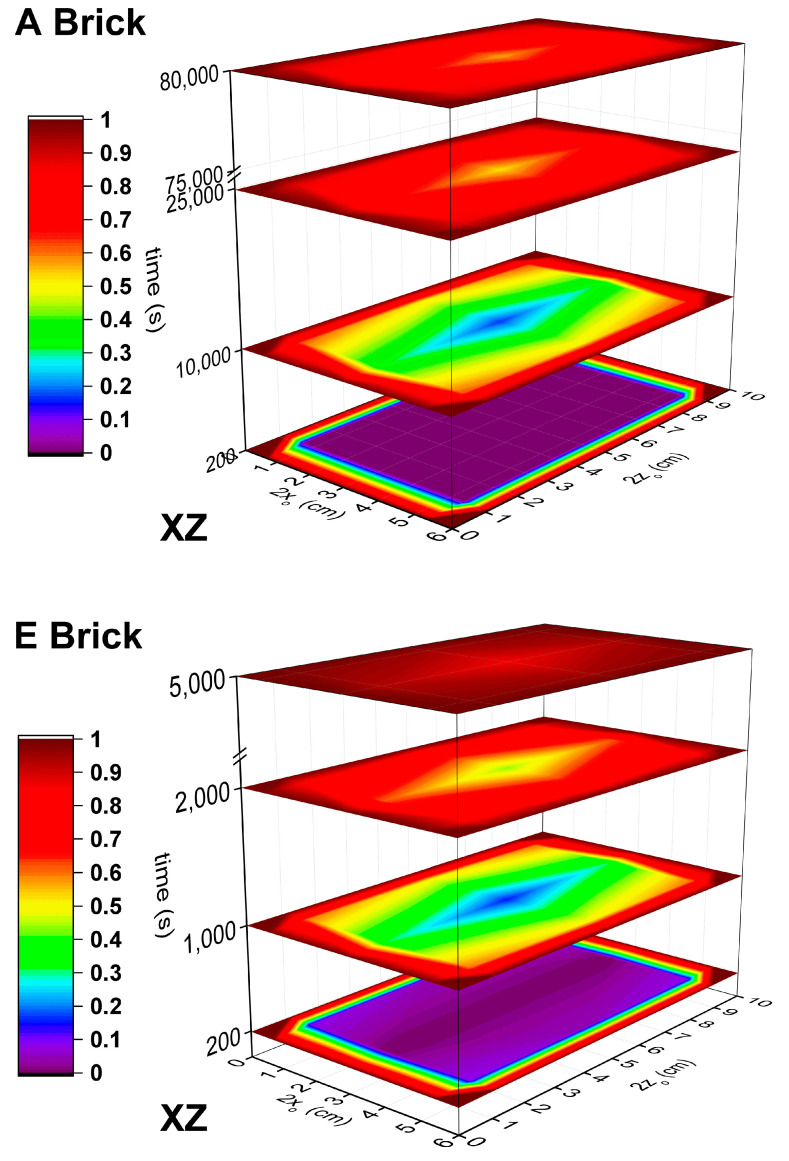
Distribution of the *sol* concentration through the bricks *A* and *E* on the XZ axis.

**Figure 9:**
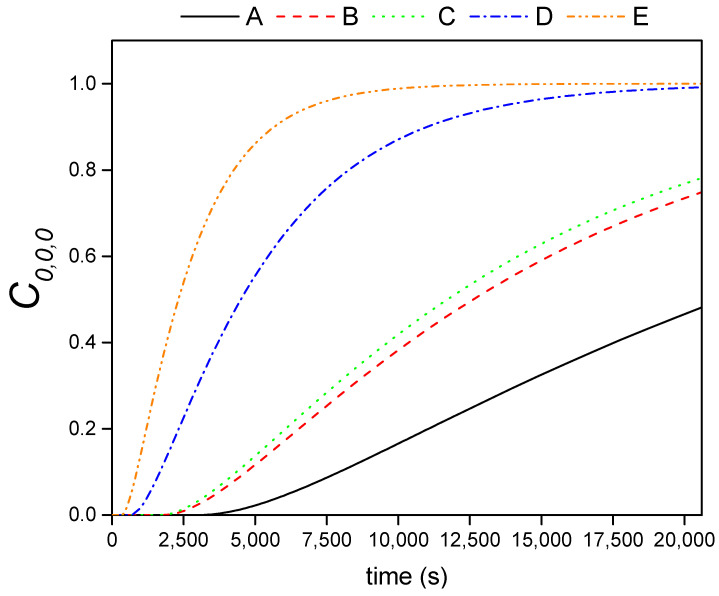
*C*_0,0,0_ profile for the studied porous bricks.

Once the proper *D_eff_*/ε coefficients have been estimated, the concentration (*C_i,k,l_*) distribution of the *sol* in the brick in a scale from 0 to 1 can be calculated for each collocation point at different times. As an example, Figure 8 displays the *sol* distribution on the *XZ* plane for the bricks of type *A* and *E* at some representative times. As shown in this figure, whereas in composite A, there predominates a *sol* concentration distribution lower than 0.5 at 10,000 s, the composite *E* needs half of that time (5000 s) to be totally saturated. It is evident that the *sol* distribution profiles decrease from the surface of the composites to their center, this point being the latest to be filled. In that way, in order to compare the filling of the different porous bricks, the evolution of the concentration in the center of the brick (*C_0,0,0_*) with time is displayed in Figure 9. From this figure, a delay in the filling of the center along with the decrease of the porosity is appreciable. Whereas the charge of *A* brick (the least porous matrix) starts at 3000 s, the most porous brick, *E*, starts at 300 s. Moreover, once the charge of the *sol* has begun, the slope of the concentration profile increases with the porosity. It can be observed that *D* and *E* bricks are able to have their center saturated below 20,000 s, a time when *A* brick has achieved only 40% of its maximum adsorption capacity. *B* and *C* bricks show similar *C*_0,0,0_ due to their similar values of *D_eff_*/ε.

It can be concluded that the proposed model perfectly fits the experimental data. Therefore, by knowing the optimal or required amount of PCM into the brick with a specific porosity, the model will allow us to calculate the immersion time needed to achieve the target.

#### 4.5.1. Influence of Porosity on *D_eff_*

**Figure 10:**
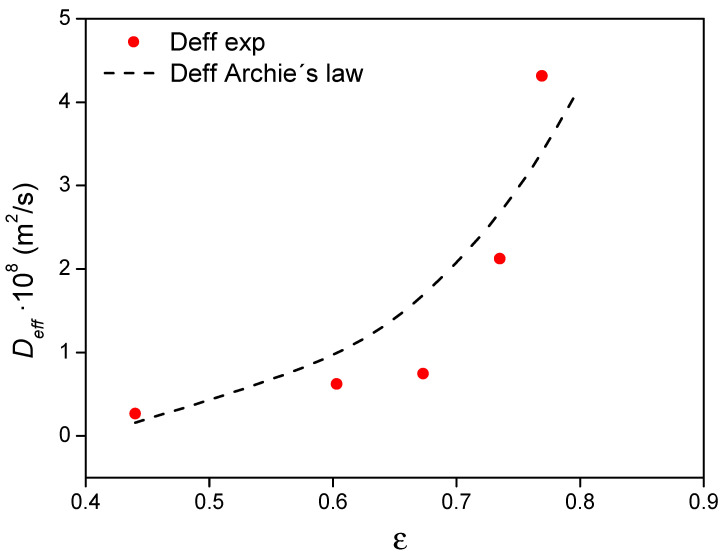
Diffusion coefficients (*D_eff_*) as a function of ε and their comparison to those obtained based on Archie´s law.

The effective diffusion coefficient of a porous media can also be estimated as a function of the porosity according to Archie´s law [49]:

(21) $$D_{eff}=D_{o}\times \varepsilon^{m}$$

where *D_o_* is the free diffusion coefficient, ε is the effective porosity, meaning all porosity that is connected and available for fluid transport, and *m* is the cementation exponent. Considering the previous expression and the known brick porosities and *D_eff_*/ε, the parameters *D_o_* and *m* were estimated. Figure 10 depicts the *D_eff_* values derived from Archie´s law, obtaining 1.424 × 10^−7^ m^2^/s as *D_o_* and 5.48 for the cementation exponent (value of the cementation exponent which is in agreement with literature for diffusive transport of water in clays, 5.4) [50].

It is plain to see that the higher the porosity, the higher the *D_eff_*, this increment being more abrupt for composite porosities larger than 0.7. According to the results, only one *D_o_* parameter can be used to predict the value of *D_eff_* as function of the porosity by means of the commented expression. The obtained *D_eff_* are lower than the diffusion coefficients of water in bricks previously reported by van der Zanden [51] as a result of the higher viscosity of the *sol* compared to water. However, the *m* and *D_o_* values indicated that the fluidity of the *sol* into the porous brick is very high and comparable to that of water and that the gelling step does not take place during the time required for the kinetic adsorption.

#### 4.5.2. Influence of the PEG-SiO_2_ Incorporation on the Composite Thermal Behavior

The thermal behavior of the empty and saturated bricks was studied to confirm the improvement of their TES capacities with the PEG-SiO_2_ incorporation. Bricks A and E were selected for this study since they presented the lowest and the highest porosities and, thus, the influence of the porosity on the TES capacity can also be analyzed. 

**Figure 11:**
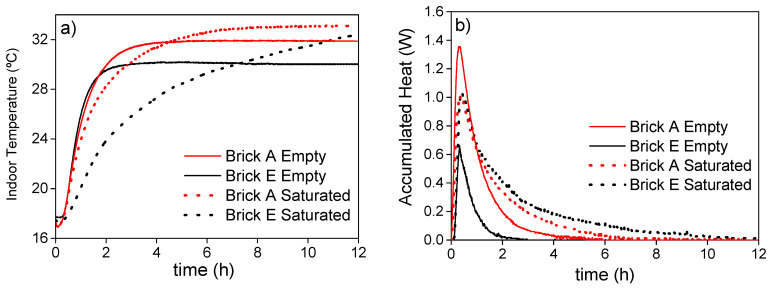
Indoor temperature (*T*_up)_ (**a**) and accumulated heat (**b**) of brick type E for the empty and saturated conditions when it is subjected to a thermal change from 15 to 42 °C.

As an example, Figure 11 shows the indoor temperature (*T*_up_) and the accumulated heat of brick type E, which presented the highest porosity, for the empty and saturated conditions when it is subjected to a thermal change from 15 to 42 °C, considering the 42 °C as the outdoor temperature. The accumulated heat (Figure 11b) is obtained by making an energy balance between the inlet and outlet heat flows.

As can be observed in Figure 11a, the PEG-SiO_2_ incorporation caused a reduction of the slope of the *T*_up_ profile at the transient state, since the energy is being accumulated by the melting of the active PEG from the PEG-SiO_2_. This slope decrease is more abrupt in the case of the brick E. This way, the indoor temperature of the saturated samples remains below that of the empty one for 4 and 7.4 h for the bricks A and E, respectively. This time is long enough to maintain a soft temperature for a whole operational day in the case of the brick E, since this comfort time is dependent on the total accumulated heat, as shown in Figure 11b. In this figure, it is observed that the minimum thermal energy storage (TES) capacity is exhibited by the empty brick E. Nevertheless, this brick becomes the most energetic one when it is filled with the PEG-SiO_2_. Thus, the prepared PEG-SiO_2_-saturated brick E with a thickness of 3 cm would be able to maintain the indoor temperature of the building below 30 °C for more than 7 h when the outdoor temperature is 42 °C, while the empty brick would reach that temperature in just one hour. In addition, it is observed how the indoor temperature of empty bricks reach the steady state faster whereas the indoor temperatures of both filled bricks require a long time to achieve the steady state. On the other hand, they overpass the temperatures of the empty ones, indicating that the substitution of air by PEG-SiO_2_ into the porous matrix of the bricks enlarges the thermal conductivity of the bricks, favoring the further energy release for restoring the accumulating energy capacity.

**Figure 12:**
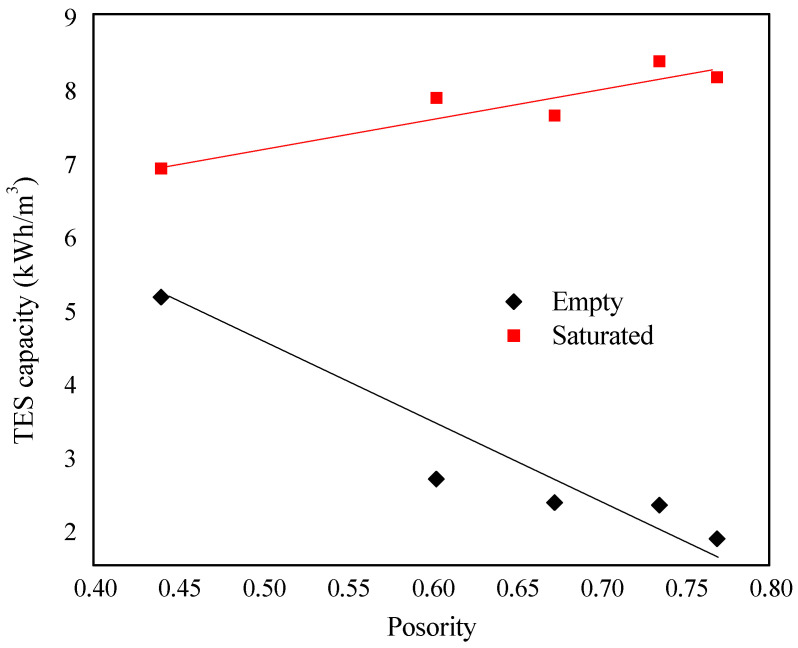
Influence of porosity on the thermal energy storage (TES) capacity of empty and saturated bricks.

Figure 12 shows the TES capacity of all bricks, determined from the accumulated heat fluxes and using Equation (2).

As expected, the higher the porosity, the lower the TES capacity of the conventional bricks (empty bricks). However, the higher the porosity, the higher the amount of the PEG-SiO_2_ in the bricks, thus increasing the TES capacity. It can also be stated that the relationship of both properties was lineal. On the other hand, comparing the improvement of the TES capacity of bricks A and E, corresponding with the lowest and the highest porosity, the increase of TES capacity was 34% and 338%, respectively. Hence, the thermal comfort of a house can be improved by using facing bricks containing shape-stabilized PEG-SiO_2_ and this comfort is strongly dependent on the initial porosity of the brick.

## 5. Conclusions

The long gelation time and low initial viscosity of the proposed PEG-SiO_2_ in the *sol* form allowed the preparation of new form-stable PCMs. Form-stable PCMs containing within 15–110 wt.% of PEG-SiO_2_ with respect to the initial dried mass of brick have been obtained depending on the brick porosity.

The kinetic adsorption studies of PEG-SiO_2_ on bricks were perfectly reproduced by means of Fick´s second law. The numerical method strategy for solving the partial differential equation problem allowed us to obtain a good fitting between experimental and theoretical data, founding *D_eff_* as the unique unknow fitting parameter. Moreover, it is possible to use only one *D_o_* coefficient to characterize the diffusion phenomena. The obtained values of the parameters D*_eff_* and *m* are compatible with those of water adsorption, indicating that this is an easy way to produce form-stable PCMs using this PEG-SiO_2_ and bricks without requiring any mechanical energy.

Contrary to empty bricks, the TES capacity of saturated bricks increased linearly with the porosity, observing a total increment of 34% and 338%, for the TES capacity of bricks A and E, respectively. The improvement in the TES capacity allowed us to maintain indoor temperatures lower than those reached by the conventional ones over a long time but as a function of the PEG-SiO_2_ content. The saturated brick E having a thickness of 3 cm kept the indoor temperature below 30 °C for more than 7 h when the outdoor temperature was 42 °C, while the empty brick reached the steady state in just one hour.

## Figures and Tables

**Table 1:** Porosity of the different brick samples.

Sample	A	B	C	D	E
**ε**	0.440	0.603	0.673	0.735	0.769

**Table 2:** Array of orthogonal collocation points and Matrix Bm for an asymmetric planar geometry, *j* and *i* being counters that indicate the contribution from each collocation point to a specific one, respectively.

Roots of Polynomials	*B_m ij_*				
0.0000	84.0000	−122.0631	58.6666	−44.6035	24.0000
0.1127	53.2379	−73.3333	26.6667	13.3333	6.7621
0.5000	−6.0000	16.6667	−21.3333	16.6667	−6.0000
0.8872	6.7621	−13.3333	26.6666	73.3333	53.2379
1.0000	24.0000	−44.6035	58.6666	−122.0632	84.0000

**Table 3:** Effective diffusion coefficients of the *sol* per porosity of each type of brick (95 per cent confidence interval).

Sample	*D_eff_*/ε (m^2^/s)
A	6.086 × 10^−9^ ± 2.343 × 10^−9^
B	1.030 × 10^−8^ ± 4.314 × 10^−9^
C	1.109 × 10^−8^ ± 5.165 × 10^−9^
D	2.887 × 10^−8^ ± 8.359 × 10^−9^
E	5.609 × 10^−8^ ± 2.202 × 10^−8^

## Data Availability

The data presented in this study are available on request from the corresponding author. The data are not publicly available due to are property of the authors.
